# The Role of the Hippocampus in Avoidance Learning and Anxiety Vulnerability

**DOI:** 10.3389/fnbeh.2014.00273

**Published:** 2014-08-08

**Authors:** Tara P. Cominski, Xilu Jiao, Jennifer E. Catuzzi, Amanda L. Stewart, Kevin C. H. Pang

**Affiliations:** ^1^Department of Neurology and Neurosciences, Rutgers – New Jersey Medical School, Rutgers, The State University of New Jersey, Newark, NJ, USA; ^2^Neurobehavioral Research Laboratory, Veterans Affairs Biomedical Research Institute, East Orange, NJ, USA; ^3^Neurobehavioral Research Laboratory, Veterans Affairs New Jersey Health Care System, East Orange, NJ, USA

**Keywords:** hippocampus, avoidance, PTSD, anxiety, WKY, synaptic plasticity, LTP

## Abstract

The hippocampus has been implicated in anxiety disorders and post-traumatic stress disorder (PTSD); human studies suggest that a dysfunctional hippocampus may be a vulnerability factor for the development of PTSD. In the current study, we examined the effect of hippocampal damage in avoidance learning, as avoidance is a core symptom of all anxiety disorders. First, the effect of hippocampal damage on avoidance learning was investigated in outbred Sprague Dawley (SD) rats. Second, the function of the hippocampus in Wistar-Kyoto (WKY) rats was compared to SD rats. The WKY rat is an animal model of behavioral inhibition, a risk factor for anxiety, and demonstrates abnormal avoidance learning, marked by facilitated avoidance acquisition and resistance to extinction. The results of the current study indicate that hippocampal damage in SD rats leads to impaired extinction of avoidance learning similar to WKY rats. Furthermore, WKY rats have reduced hippocampal volume and impaired hippocampal synaptic plasticity as compared to SD rats. These results suggest that hippocampal dysfunction enhances the development of persistent avoidance responding and, thus, may confer vulnerability to the development of anxiety disorders and PTSD.

## Introduction

The development of post-traumatic stress disorder (PTSD) and anxiety disorders is a function of an individual’s experience and inherent vulnerability. While much research effort has been devoted to the effects of traumatic stress on individuals, less effort has been devoted to the study of vulnerability factors. Vulnerability or risk factors may be inherited (i.e., personality traits or genetic variations) or due to prior experiences (i.e., abuse or experience of a previous trauma).

The hippocampus is a brain region implicated in PTSD. Patients with PTSD have reduced hippocampal volume (Gurvits et al., 1996; Villarreal et al., 2002). A recent study, using high resolution MRI, showed that reduced hippocampal volume in PTSD patients is localized to the CA3/DG region of the hippocampus (Wang et al., 2010). These findings agree with animal studies that showed severe chronic stress leads to atrophy of apical dendrites in the CA3 region, reduced neurogenesis, and mature granule cell death in the dentate gyrus (DG) of the hippocampus due to elevated levels of glucocorticoids (Gould et al., 1990, 1998; McEwen et al., 1995; Gould and Tanapat, 1999). Based on these studies, it was hypothesized that reduced hippocampal volume in individuals with PTSD was a consequence of the traumatic event and subsequent development of PTSD (Bremner, 2001). However, more recent human research has challenged this hypothesis.

Rather than a consequence of the traumatic experience, reduced hippocampal volume may be a risk factor for developing PTSD. The first suggestion of this was a study of identical twins discordant for combat experience (Gilbertson et al., 2002). In this study, individuals with combat experience were divided into those diagnosed with PTSD and those not diagnosed and then paired with their twin siblings who were not exposed to combat and were not diagnosed with PTSD. The results of this study replicated the previous finding of reduced hippocampal volume in combat-exposed individuals with PTSD compared to combat-exposed individuals without PTSD (Gurvits et al., 1996). Importantly, the twin sibling of the combat-exposed PTSD subject had reduced hippocampal volume compared to the twin sibling of the combat-exposed non-PTSD subjects. Thus, these data suggested that decreased hippocampal volume pre-existed trauma exposure and diagnosis of PTSD. A subsequent study linked the reduced hippocampal volume to a learning impairment (Gilbertson et al., 2007). Therefore, the evidence suggests that reduced hippocampal volume, with concomitant dysfunction, is a risk factor for PTSD. Despite this relationship, the manner in which hippocampal dysfunction contributes as a risk factor for PTSD is unclear.

Excessive avoidance is a core feature of all anxiety disorders and is a core component of PTSD diagnosis (American Psychiatric Association, 2013). Moreover, pathological avoidance symptoms increase with time after a trauma and parallel the trajectory of PTSD (O’Donnell et al., 2007). Once developed, avoidant responses are notoriously difficult to treat, and are resistant to pharmacological and cognitive behavioral therapy. The growth of avoidance suggests a learning component to the pathological avoidance. Thus, knowledge of the mechanisms involved in avoidance learning may lead to important insights into the development of avoidance symptoms in anxiety disorders and PTSD.

Although the role of the hippocampus in anxiety-related behaviors like elevated plus maze and fear conditioning has been studied extensively, its role in active avoidance behavior is not well established [for review Barkus et al. (2010)]. An abnormal hippocampus may provide risk to the development of anxiety disorders and PTSD by enhancing sensitivity to active avoidance behaviors. Hippocampal damage leads to facilitated avoidance learning in shuttle avoidance [for review, see Olton (1973) and Black et al. (1977)] and lever-press avoidance (Schmaltz and Giulian, 1972). In addition, we previously showed that damage of GABAergic neurons in the medial septum, a major non-cortical input to the hippocampus, prior to avoidance training impaired extinction but not acquisition of the avoidance response (Pang et al., 2011). Thus, dysfunction of the hippocampus may enhance the rate of avoidance acquisition and the development of persistent avoidant responding, thereby resulting in risk for anxiety disorders and PTSD.

The Wistar-Kyoto (WKY) rat is an animal model of behavioral inhibition and displays many characteristics related to anxiety disorders. Trait behavioral inhibition is a vulnerability factor for the development of anxiety disorders, as behaviorally inhibited children are more likely to develop anxiety disorders (Kagan et al., 1987). WKY rats demonstrate the trait behavioral inhibition phenotype, observed as decreased activity and withdrawal in novel social (Pare, 2000) and non-social challenges (Pare, 1994). WKY rats display low activity in the open field (Pare, 1994) and have enhanced sensitivity to stress-induced ulcer formation (Pare, 1989), hyper-responsive peripheral and central stress responses (Pardon et al., 2002), and learning and memory alterations (Ferguson and Cada, 2004). Of particular relevance to this study, WKY rats acquire lever-press avoidance faster and to a higher degree than Sprague Dawley (SD) rats (Servatius et al., 2008). Avoidant behaviors of WKY rats are also more persistent during extinction training than in SD rats, especially at high shock intensity (Jiao et al., 2011). In fact, extinction following avoidance learning at high shock intensity was virtually non-existent in WKY rats, a pattern that was not displayed by SD rats.

The present study was performed to further elucidate the role of the hippocampus in acquisition and extinction of lever-press avoidance using two approaches. First, the effect of hippocampal damage on avoidance learning was investigated in outbred SD rats. Second, hippocampal synaptic plasticity and hippocampal volume were assessed in WKY rats since human studies suggested impaired hippocampal function in those individuals with vulnerability for PTSD. The results of the current study suggest that a dysfunctional hippocampus enhances the development of persistent avoidant responses.

## Materials and Methods

### Subjects

Male SD rats (*n* = 43) were 300–350 g and male Wistar-Kyoto (WKY, *n* = 8) rats were 200–250 g at the start of the behavioral study. Thirty-five SD rats underwent surgery for lesions or sham procedures. Eight SD and eight WKY rats were behaviorally tested without surgery. All rats were housed individually on a 12-h light/dark cycle with lights turning on at 7:00 a.m. Training and testing were performed during the light phase of the light/dark cycle. All procedures were conducted in accordance with the NIH Guide for the Care and Use of Laboratory Animals and were approved by the IACUC of the Veterans Affairs Medical Center at East Orange, New Jersey.

### Lesion surgery

**Figure 1:**
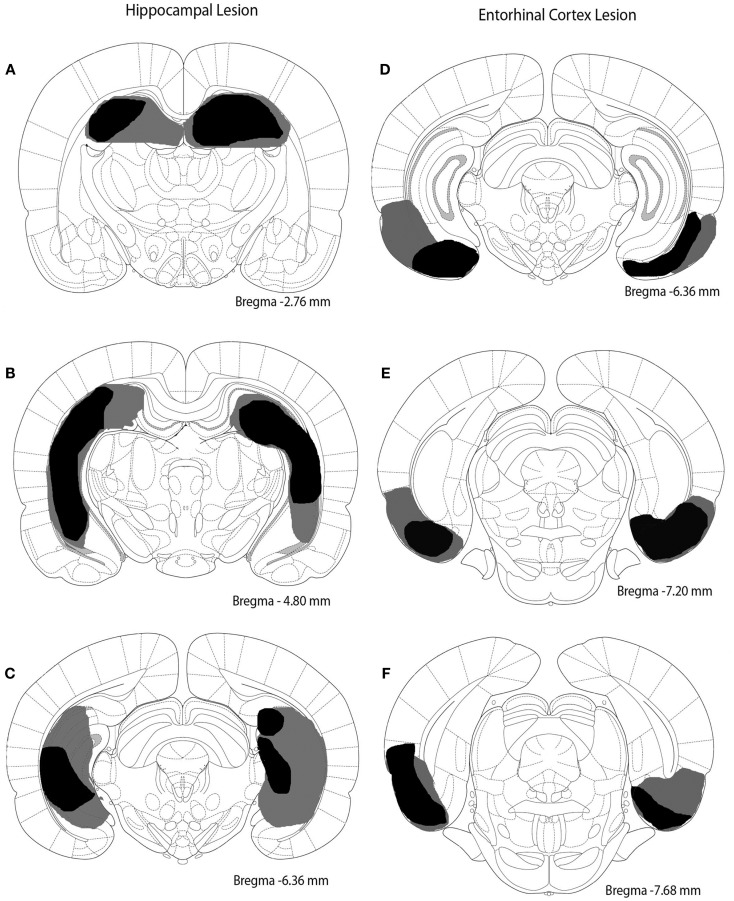
**Excitotoxic lesions of the hippocampus and entorhinal cortex**. The black shaded regions indicate the smallest lesion observed and gray shaded regions indicate the largest lesion observed. **(A–C)** depict the hippocampal lesion at three different anterior–posterior locations (approximately -2.76, -4.80, and -6.36 mm from bregma). **(D–F)** represent the entorhinal cortex lesion at three different anterior–posterior sites (approximately -6.36, -7.20, and -7.68 mm from bregma).

Rats were anesthetized with isoflurane (2%). Burr holes were drilled into the skull overlying the hippocampus or entorhinal cortex. The coordinates (in mm) for the entorhinal cortex lesion sites in relation to bregma were as follows (four sites per hemisphere): AP -5.3, ML ±6.5, DV -5.0; AP -6.0, ML ±6.5, DV -5.0; AP -6.7, ML ±5.0, DV -6.5; AP -7.4, ML ± 5.0, DV -6.5. The coordinates for the hippocampal lesion sites in relation to bregma were as follows (five sites per hemisphere): AP -2.5, ML ±1.6, DV -3.8; AP -4.2, ML ±2.6, DV -3.1; AP -5.3, ML ±4.4, DV -3.4; AP -5.8, ML ±5.6, DV -4.1; AP -6.0, ML ±5.6, DV -4.1. Injections were made bilaterally. The needle of a Hamilton syringe was inserted into the desired location to infuse saline for sham surgery or ibotenic acid (10 μg/μl) to damage hippocampus or entorhinal cortex. Infusions occurred at a rate of 0.1 μl/min with a volume of 0.5 μl dispensed per site. Rats were allowed at least 10 days to recover from surgery. Extent and location of lesions are depicted in Figure 1.

### Behavior

#### Avoidance learning

Rats were trained in an operant box with a lever (10.5 cm above the floor), a cue light (20.5 cm above the grid floor), and a speaker (26 cm above the grid floor) on one wall and a light (14 W) on the opposite wall that was constantly lit during the session. Scrambled footshocks were delivered through the grid floor (Coulbourn Instruments, Langhorn, PA, USA). The operant box was enclosed in a sound-attenuating box.

Avoidance learning occurred as described previously (Pang et al., 2011). Briefly, each session was separated by 2–3 days (3 sessions/week). Each session began with a 60-s stimulus-free period, followed by 20 trials. A trial started with the presentation of the warning signal (1000-Hz 75-dB tone, 10 dB above background noise). A lever response made during the first 60 s of the trial immediately terminated the warning signal and initiated 3-min intertrial interval (ITI). This response was an avoidance response, as the rat avoided the footshock. If an avoidance response was not made, foot shocks (2 mA, 0.5 s duration, 3 s intershock interval) were delivered starting at 60 s and continued until a lever response was made (scored as an “escape response”) or 100 shocks were delivered. Immediately following an escape response or the maximum number of foot shocks, the warning signal was terminated and a 3-min ITI was initiated. All ITIs were signaled by a flashing light (ITI signal, 5 Hz, 50% duty cycle). Responses during the ITI had no effect but were recorded. The acquisition phase consisted of 12 sessions.

During the extinction phase, all procedures were the same as in the acquisition phase except the foot shock and ITI signal were omitted. Although shocks were omitted, responses during the first 60 s of the trial were designated as “avoidance” responses, and those with latencies greater than 60 s were designated as “escape” responses. The extinction phase consisted of six sessions.

#### Data analysis

Data are expressed as mean ± standard error of the mean. Performance was assessed by calculating the proportion of trials in each session with an avoidance response. A mixed design ANOVA with session as the within subject factor and lesion/strain as the between subjects factor was performed. All lesion groups and unoperated SD and WKY groups were included in this overall ANOVA and are captured in the lesion/strain factor. Separate analyses were performed for the acquisition and the extinction phases. To determine whether non-specific responding might be increased by lesion or strain, lever presses during each minute of the ITI were analyzed. Mean lever presses per trial per minute was determined for the ITI and assessed statistically using a mixed design ANOVA with session and ITI minute as within subject factors and lesion or strain as a between subjects factor. Statistical analysis was performed with α = 0.05 using SPSS for Windows (version 12.0.1, SPSS, Inc., Chicago, IL, USA). Mixed design analysis of variance (ANOVA) was used to compare groups. Mauchly’s test was used to determine violations in the assumptions of sphericity for repeated measure factors and Greenhouse–Geisser correction was used in the appropriate situations to correct for violations (Geisser and Greenhouse, 1958). Corrected statistics are only reported when the uncorrected and corrected *p*-values disagreed with respect to significance; otherwise only the uncorrected values are reported. Tukey’s *post hoc* tests were performed to specify group differences. Analysis of covariance (ANCOVA) was performed to determine whether significant effects in extinction remained after covarying performance on the last acquisition session. Interactions were evaluated by *F*-test.

### Electrophysiology

#### Recording

Six naïve SD and six WKY male rats were obtained from Harlan Laboratories (Indianapolis, IN, USA) at three months of age. All rats were given at least one week to acclimate to the new surroundings prior to recordings. Recordings were performed during the light phase of the light/dark cycle. Procedures were as described previously (Yoder and Pang, 2005). Rats were anesthetized with urethane (1.5 g/kg, i.p.) and immediately placed in a stereotaxic apparatus. A recording electrode (75 μm, Teflon coated stainless steel wire) was placed in the hilar region of the DG (AP 4.0 mm posterior and 2.5 mm lateral from bregma, 2.8–3.2 mm ventral from the brain surface; WKY rats: 4.0 mm posterior, 2.8 mm lateral from bregma, 2.8–3.2 mm ventral from the brain surface) and a stimulating electrode (125 μm, Teflon coated stainless steel wire) was inserted into the medial perforant pathway (mPP) (SD rats: 8.1 mm posterior and 3.1 mm lateral from bregma, 2.0–2.8 mm ventral from brain surface; WKY rats: 8.1 mm posterior and 3.6 mm lateral from bregma, 2.0–2.8 mm ventral from brain surface). The response was optimized within the dorsal/ventral coordinate range specified. Constant current stimulation (biphasic pulse, 300μs duration; AM Systems Isolated Pulse Stimulator, Model 2100, Carlsborg, WA, USA) was applied to the mPP at a rate of 1/10 s. Evoked field potentials were recorded from the DG after amplification of 1000× and bandpass filtering between 0.1 Hz and 5 KHz (AM Systems Differential AC Amplifier, Model 1700, Carlsborg, WA, USA). Evoked responses were visualized on a digital oscilloscope and off-line analysis was performed using SciWorks software (version 7.2 SP1, DataWave Technologies).

A total of six input–output (i/o) response curves were generated to monitor changes in slope of field EPSP (fEPSP) and population spike amplitude (leading peak to valley) before and after high frequency stimulation (HFS). The i/o curves were generated using 100–1000 μA stimulation. Average waveforms were generated from five evoked responses at each stimulus intensity, and slopes of fEPSP and population spike were measured from these averaged waveforms. Two i/o curves were used to establish baseline. HFS to induce LTP was based on parameters established previously (Messaoudi et al., 2002). Stimulation for HFS was delivered at the lowest intensity that generated the maximal population spike for each animal and consisted of three sets of four trains, each train consisted of eight pulses given at a frequency of 400 Hz, intertrain interval 10 s, and interset interval of 5 min. Early phase LTP was determined from averaged i/o curves at 15 min and 1 h after HFS. Late phase LTP was evaluated from averaged i/o curves generated at 2 and 3 h after HFS. At the end of the recording session, small lesions were made to mark electrode placement (30 s, 500μA) and brains were processed as described below.

#### Statistical analysis

Data are expressed as mean ± standard error of the mean. Statistics were performed on raw values of fEPSP slope and population spike amplitude determined from averaged waveforms. fEPSP slope and population spike amplitude were each assessed separately for each strain using a 3 (phase) × 2 (time) × 7 (stimulus Intensity) repeated measures ANOVA. Statistical analysis was performed with SPSS similar to that described for the behavioral studies.

### Histology

At the end of behavioral testing or recording, all animals were perfused intracardially with saline followed by formalin. Brains were extracted and submerged overnight in formalin followed by 30% sucrose. Brains were sectioned (50 μm) through the hippocampus and entorhinal cortex with a sliding microtome. Sections were stained with cresyl violet. For the lesion study, location and extent of brain damage were assessed (Figure 1). For the electrophysiology study, placement of the electrode tips was confirmed under a light microscope.

In a separate group of rats, volumetric comparisons were made between SD and WKY rats. Rats (*n* = 5 for each strain) were sacrificed, perfused, and brains extracted. Brains were sectioned at 50 μm thickness and every fifth section was collected and stained with cresyl violet. Volume of the hippocampus, neocortex, corpus callosum, and striatum was estimated using the Cavalieri method (Slomianka and West, 2005) (StereoInvestigator, v 7.0, MicroBrightField, Colchester, VT, USA). A MANOVA was used to compare volumes of the various brain regions between strains (SPSS for Windows).

## Results

### Avoidance acquisition

#### Avoidance responses

**Figure 2:**
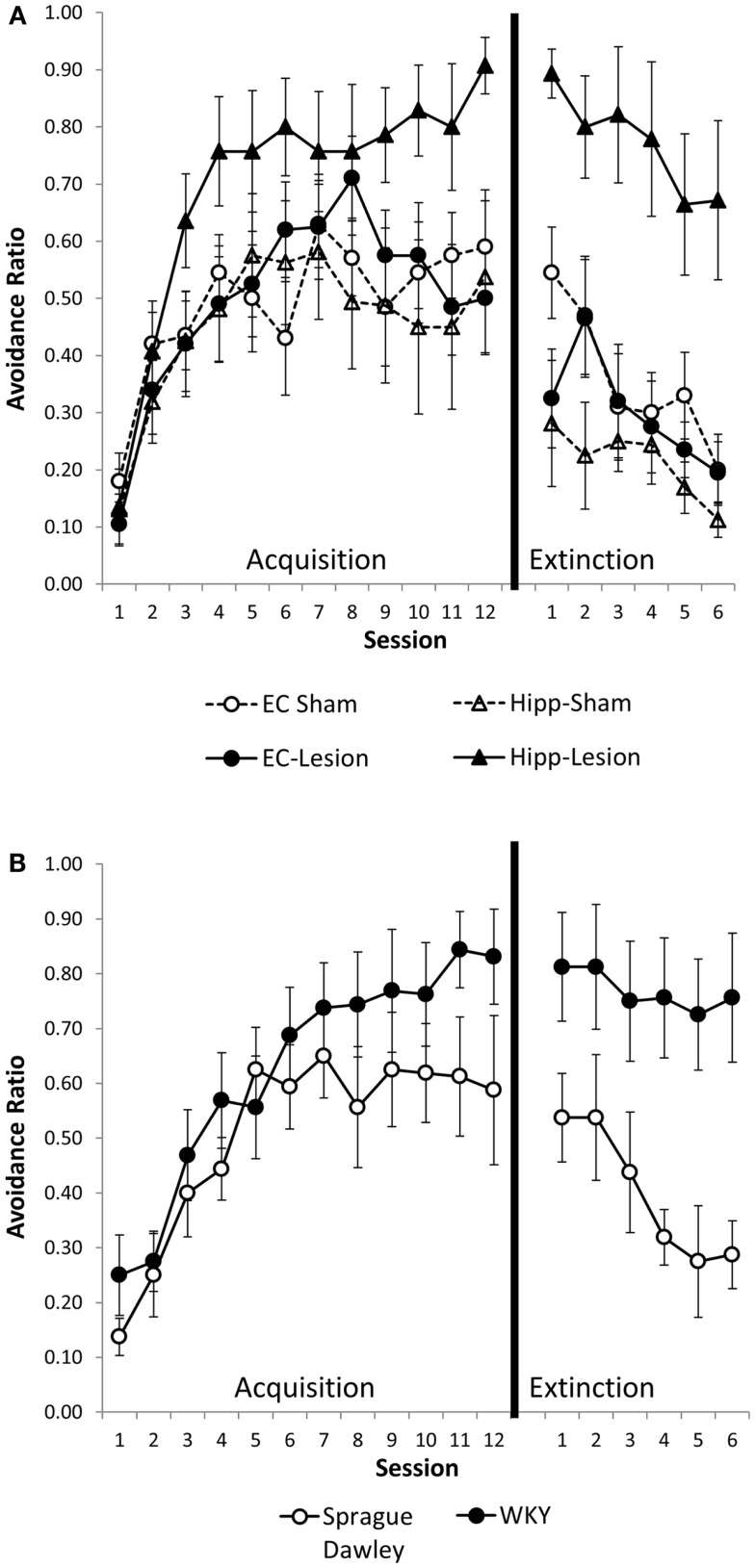
**Avoidance acquisition and extinction following hippocampal and entorhinal cortex lesion and in WKY rats**. Hippocampal and entorhinal cortex lesions did not alter avoidance acquisition **(A)**. Rats with hippocampal lesions were impaired in extinction learning compared to sham controls **(A)**. Acquisition of avoidance in WKY rats did not differ from SD rats **(B)**. WKY rats exhibited a trend toward impaired extinction of avoidant responding **(B)**. Although all six groups were statistically analyzed together, lesion **(A)** and unoperated **(B)** groups are displayed separately for clarity.

Hippocampal and entorhinal cortex lesions did not alter acquisition of avoidance (Figure 2A). Similarly, acquisition of avoidance in WKY rats did not differ from SD rats (Figure 2B). Rats from all groups increased avoidance responding with training [Figure 2; main effect of session: *F*(11,495) = 30.55, *p* < 0.001]. Acquisition of avoidance did not differ between lesion groups nor between strains [main effect of lesion/strain, *F*(5,45) = 1.91, *p* = 0.111; session × lesion/strain interaction, *F*(55,495) = 1.14, *p* = 0.237] (Figures 2A,B).

#### ITI responses

**Figure 3:**
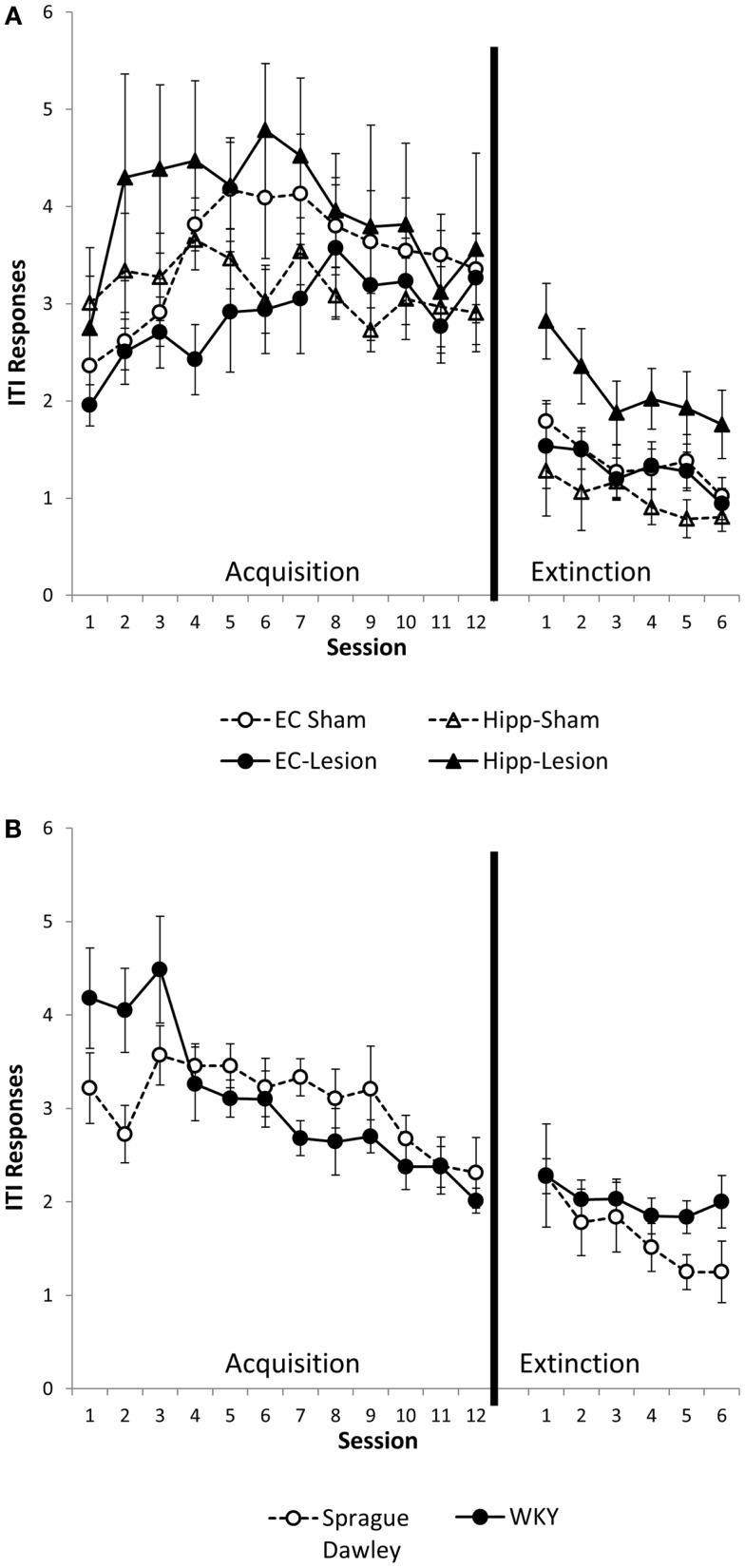
**ITI responding during the first minute of the intertrial interval (ITI)**. Hippocampal lesion increased ITI responding during the first minute of the ITI during acquisition and extinction **(A)**. Group differences were not present during the second or third minute of the ITI (not shown). WKY rats showed a trend for increased ITI responding during the first minute of the ITI in the avoidance phase, but not extinction **(B)**. Strain differences were not observed during the second or third minute of the ITI (not shown).

Intertrial interval responses were analyzed because they represent non-reinforced responses. The number of ITI responses generally increased with training, peaking around session 4 or 5, then leveling off [main effect of session, *F*(11,484) = 6.02, *p* < 0.001]. ITI responding was greater in the first minute of the ITI as compared to the second or third minutes [main effect of ITI, *F*(2,88) = 871.10, *p* < 0.001]. Whereas the main effect of lesion/strain [*F*(1,44) = 1.6, *p* = 0.18] was not significant, the lesion/strain × session × ITI interaction [*F*(110,968) = 1.54, *p* = 0.001] did reach significance. In *post hoc* analysis of each ITI minute, lesion/strain affected the first minute ITI response [lesion/strain × session interaction, *F*(55,495) = 1.78, *p* = 0.001; main effect of lesion/strain, *F*(5,45) = 1.62, *p* = 0.174] (Figure 3), but not second or third minute ITI responses [main effects, *F*(5,45) ≤ 1.61, *p* > 0.171; lesion/strain × session interaction, *F*(55,495) ≤ 1.17, *p* > 0.196]. Comparisons made between sham and lesions and between unoperated SD and WKY rats for first minute ITI responses revealed that hippocampal but not entorhinal lesions facilitated responding [main effect, *F*(1,13) = 8.32, *p* = 0.013], and strain differences trended toward significance with WKY tending to make more ITI responses than SD rats [session × strain interaction, *F*(11,154) = 2.42, corrected *p* = 0.051]. Thus, hippocampal but not entorhinal cortex lesions increased ITI responding during the first, but not second or third, minutes of the ITI (Figure 3A). A similar trend was present for WKY rats as compared to SD rats (Figure 3B).

### Avoidance extinction

#### Avoidance responses

Hippocampal lesion and WKY rats were impaired in extinction of avoidance responding (Figures 2A,B). Overall, rats decreased avoidance responding during extinction training, [*F*(5,225) = 13.73, *p* < 0.001]. Lesion/strain differed [*F*(1,45) = 9.09, *p* < 0.001] but session did not interact with lesion/strain [*F*(25,225) = 1.12, *p* = 0.322]. *Post hoc* analysis demonstrated that rats with hippocampal lesions and WKY rats extinguished their avoidant responding slower than the other groups (*p* < 0.05) (Figures 2A,B). Moreover, extinction of hippocampal lesion and WKY rats were not different. The rate of extinction can be affected by performance immediately prior to extinction training. Because groups differed in their asymptotic level of avoidance performance at the end of acquisition, extinction was analyzed with performance on session 12 of acquisition as a covariate. Even after covarying performance on session 12, main effects of lesion and strain still differed [*F*(5,44) = 5.71, *p* < 0.001], demonstrating persistent avoidant responding in rats with hippocampal lesions and WKY rats but not rats with entorhinal cortex lesions.

#### ITI responses

Similar to the acquisition phase, ITI responses during extinction were greater during the first minute of ITI as compared to the second and third minutes [main effect of ITI, *F*(2,90) = 306.47, *p* < 0.001]. ITI responses were most numerous during early extinction sessions and gradually decreased with extinction training [main effect of session, *F*(5,225) = 13.75, *p* < 0.001] (Figures 3A,B). Lesion/strain interacted with ITI, [*F*(10,90) = 2.53, *p* = 0.01], but did not interact with session, [*F*(25,225) = 0.60, *p* = 0.934]. The triple interaction did not reach significance, [*F*(50,450) = 2.94, *p* = 0.058]. Upon further analysis, it was determined that lesion/strain was significantly different during the first minute of ITI, [*F*(5,44) = 3.249, *p* = 0.014] (Figures 3A,B). *Post hoc* analysis revealed a significant group difference between hippocampal lesion and hippocampal sham during the first minute (Figure 3A). During the third minute of ITI, lesion/strain was also different, [*F*(5,11) = 2.44, *p* = 0.048]; however, *post hoc* analysis found no significant group differences. Thus, hippocampal lesions increased ITI responding during the first, but not second or third, minute of the ITI (Figure 3A).

To summarize, hippocampal lesions slowed extinction of avoidant responses similar to that observed with WKY rats (Figures 2A,B). Moreover, non-reinforced ITI responding (during minute one) was increased in hippocampal lesion and WKY rats (Figures 3A,B). These effects were not observed following damage to the entorhinal cortex.

### Hippocampal volume

**Figure 4:**
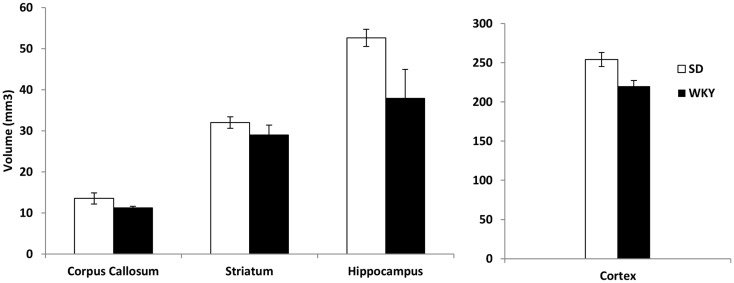
**The volume of the hippocampus and cortex was significantly reduced in WKY compared to SD rats**. In contrast, strain differences were not observed for striatum and corpus callosum.

Because SD rats with hippocampal damage mimicked the persistent avoidant behaviors of WKY rats, we investigated whether WKY rats might have an abnormal hippocampus as demonstrated by a smaller hippocampus and impaired hippocampal synaptic plasticity. Hippocampal and cortical volume was reduced in WKY rats compared to SD rats (Figure 4). The volume of the hippocampus, neocortex, corpus callosum, and striatum was estimated using the Cavalieri method. Regional brain volumes in WKY rats differed from SD rats [main effect of strain, Wilks’ Lambda, *F*(4,5) = 6.348, *p* < 0.05]. WKY rats had significantly smaller hippocampus [*F*(1,8) = 25.396, *p* < 0.01] and cortex [*F*(1,8) = 9.017, *p* < 0.05] compared to SD rats (Figure 4). Corpus callosum and striatum were not different between strains.

### Hippocampal electrophysiology

**Figure 5:**
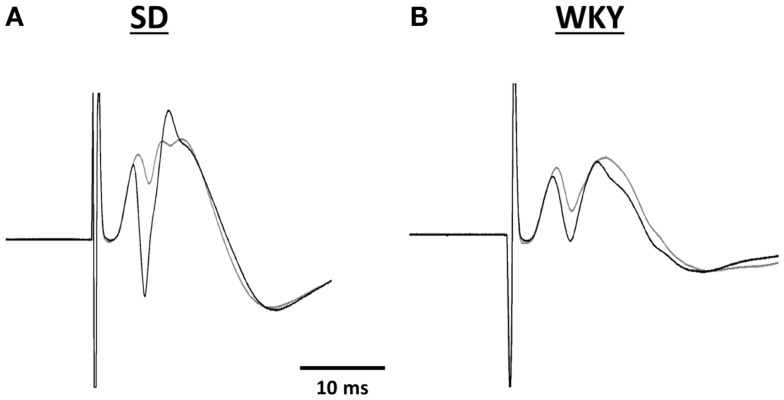
**Representative traces of evoked potentials recorded in the hilus of the dentate gyrus from SD (A) and WKY (B) rats**. Evoked potentials were in response to stimulation of the medial perforant pathway before (gray line) and 180 min after high frequency stimulation (black line).

**Figure 6:**
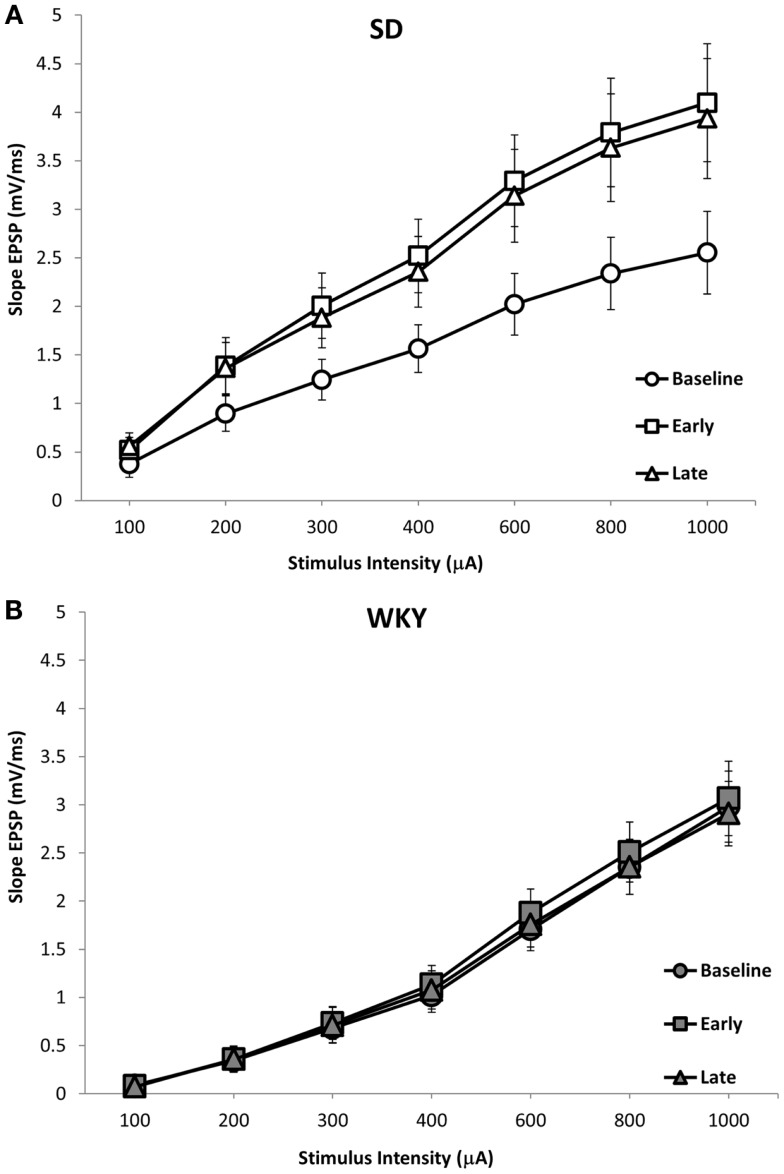
**LTP of the dentate gyrus field EPSP (fEPSP) following HFS of the medial perforant pathway in SD and WKY rats**. SD rats exhibited early and late phase LTP of the fEPSP **(A)**. In contrast, WKY rats did not demonstrate LTP at either early or late time points **(B)**. Displayed are input–output (i/o) curves showing the baseline response, early phase LTP and late phase LTP. Shown are an average of two i/o curves generated prior to HFS (baseline), an average of i/o curves generated 15-min and 1-h after HFS (early), and an average of i/o curves generated 2- and 3-h after HFS (late).

Long-term potentiation (LTP) of the mPP to DG synapse was impaired in WKY rats. Evoked field potentials had similar waveforms in SD and WKY rats (Figure 5). LTP of the fEPSP was observed in SD rats, but not in WKY rats (Figures 6A,B). In SD rats, both early phase LTP (15 min and 1 h after HFS) and late phase LTP (2 and 3 h after HFS) were observed, as main effect of phase [*F*(2, 10) = 5.229 *p* = 0.028] and the phase × stimulus intensity interaction [*F*(12,60) = 4.507, *p* < 0.001] were significant (Figure 6A). The main effect of stimulus intensity was also significant, [*F*(6,30) = 13.139, *p* < 0.001]. In contrast to SD rats, LTP of the fEPSP was not observed in WKY rats (Figure 6B). Neither main effect of phase [*F*(2,10) = 1.913, *p* = 0.198] nor the phase × stimulus intensity interaction [*F*(12,60) = 1.794, *p* = 0.07] were significant. The main effect of stimulus intensity was significant, [*F*(6,30) = 22.234 *p* < 0.001].

**Figure 7:**
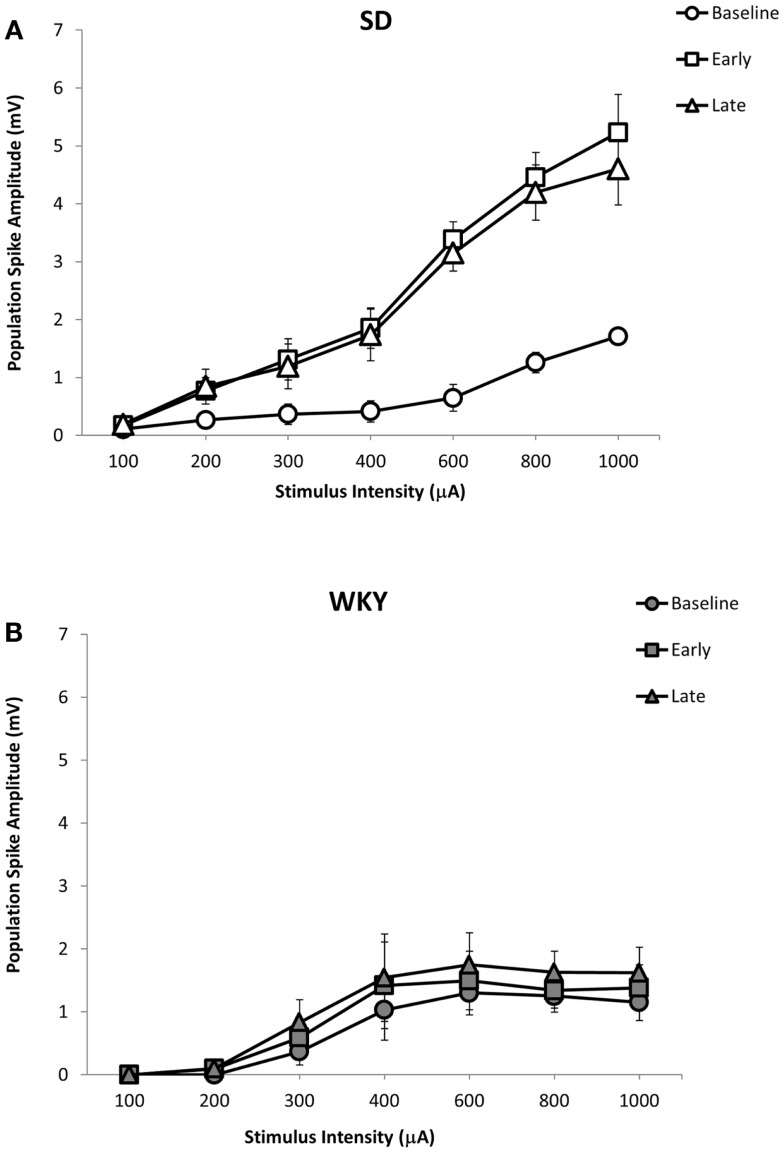
**LTP of the dentate gyrus population spike following HFS of the medial perforant pathway in SD and WKY rats**. Following HFS, SD rats exhibited robust early and late phase LTP of the population spike **(A)**. In contrast to SD rats, early or late phase LTP of the population spike was not observed in WKY rats **(B)**.

Similar to fEPSP, LTP of the population spike was observed in SD but not in WKY rats (Figures 5 and 7A,B). In SD rats, early and late phase LTP were observed (Figure 7A), as main effect of phase [*F*(2, 10) = 22.393, *p* < 0.001] and the phase × stimulus intensity interaction [*F*(12,60) = 7.014 *p* < 0.001] were significant. The main effect of stimulus intensity was also significant, [*F*(6,30) = 14.660, *p* < 0.001]. LTP of the population spike was not observed in WKY rats (Figure 7B). Neither main effect of phase [*F*(2,10) = 4.291; corrected *p* = 0.085] nor the phase × stimulus intensity interaction [*F*(12,60) = 1.543, *p* = 0.134] were significant, although the main effect of stimulus intensity was significant, [*F*(6,30) = 3.081, *p* = 0.018].

## Discussion

An abnormal hippocampus may provide risk for developing PTSD. Smaller hippocampal volume and associated poorer learning were observed in soldiers with PTSD and their non-combat, non-PTSD twin siblings (Gurvits et al., 1996; Gilbertson et al., 2002) The present study investigated whether impaired hippocampal function might enhance anxiety risk by increasing the sensitivity and persistence of avoidance learning, as avoidance is a core symptom of all anxiety disorders and PTSD (American Psychiatric Association, 2013). Our results show that hippocampal damage enhances the formation of persistent lever-press avoidance and non-reinforced responding, similar to an animal model of anxiety vulnerability, the WKY rat. Moreover, reduced hippocampal volume and impaired hippocampal synaptic plasticity were evident in the WKY rat, potentially contributing to their persistent avoidant responding.

The role of the hippocampus in lever-press avoidance learning is an understudied topic. In one study, hippocampal damage caused enhanced acquisition of lever-press avoidance (Schmaltz and Giulian, 1972). The present study found a trend for rats with hippocampal damage to acquire lever-press avoidance more rapidly and to a greater asymptotic level, although these results were not statistically reliable. The Schmaltz and Giulian study found no effect of hippocampal lesions on extinction of lever-press avoidance, which is in contrast to the results of the present study. This discrepancy can be explained by several differences between the two studies. Schmaltz and Giulian made their hippocampal lesions by aspiration after acquisition was stable. In the current study, hippocampal lesions using ibotenic acid were performed prior to the start of avoidance acquisition. Results from both studies are consistent with the view that persistent avoidant responding is set during acquisition due to abnormal learning of the avoidance response, and not specifically due to effects of hippocampal lesions on extinction learning. In addition, the shock intensity used in the Schmaltz and Giulian study was much lower than the current study. We have previously shown that shock intensity is particularly important in the persistence of avoidance responding during extinction in WKY rats (Jiao et al., 2011). Thus, the present study extends the work of Schmaltz and Giulian in elucidating the effect of hippocampal lesion on lever-press avoidance and its extinction.

In contrast to active avoidance, the role of the hippocampus in anxiety-related behaviors as assessed in behavioral tests like the elevated plus maze and fear conditioning is better characterized [for review Barkus et al. (2010)]. Complete lesions of the hippocampus and selective lesions of the ventral hippocampus lead to reduced conditioned freezing (Richmond et al., 1999). Rats with ventral hippocampal lesions, but not dorsal hippocampal lesions, enter the open arms in the elevated plus maze more freely than sham rats (Bannerman et al., 2002; Kjelstrup et al., 2002). While these results might suggest an anxiolytic nature of hippocampal damage, the effects of hippocampal damage on elevated plus maze are not always clear; they depend on the extent of hippocampal damage, location of the damage, and the dependent measure evaluated. Still, enhanced persistent avoidant responding following hippocampal damage in the present study is more indicative of anxiogenic rather than anxiolytic action. Future studies are needed to disentangle the role of the hippocampus in different symptoms and tests of anxiety.

The persistent avoidance responding of SD rats with hippocampal damage and intact WKY rats suggests that WKY rats may have an abnormal hippocampus. In order to investigate this possibility, we compared hippocampal volume in SD and WKY rats. WKY rats had reduced hippocampal and cortical volume, but similar striatum and corpus callosum volume to SD rats. The reduced hippocampal volume was similar in magnitude to soldiers with PTSD and their non-combat, non-PTSD twin siblings (Gilbertson et al., 2002). One difference between the present study and the human studies is the difference in cortical volume in WKY rats. In the human studies, cortical volume was not reported; however, total brain volume was not affected in these studies. Thus, WKY rats appear to replicate some aspects of PTSD risk factors, but there may be additional impairments exhibited by this animal model.

Combat, PTSD patients and their twin siblings had impaired configural learning that was associated with reduced hippocampal volume (Gilbertson et al., 2007). In order to determine whether the reduced hippocampal volume in WKY rats amounted to a functional impairment, we assessed hippocampal synaptic plasticity. LTP is currently the best model of the synaptic changes hypothesized to occur during learning (Morris et al., 1986). The waveform of the evoked response to stimulation of the medial perforant path was similar in SD and WKY rats. However, the lack of LTP in WKY rats following HFS was dramatic and supports the idea that impaired hippocampal synaptic plasticity may underlie the impairments in hippocampal dependent learning displayed by WKY rats (Clements and Wainwright, 2007; Clements et al., 2007). Furthermore, the impaired hippocampal synaptic plasticity in WKY rats may contribute to the persistent avoidance learning, as SD rats with damaged hippocampus behaved similarly.

WKY rats normally demonstrate enhanced acquisition of lever-press avoidance, as well as the perseveration of this response (Servatius et al., 2008; Jiao et al., 2011). In the current study, a significant difference was not found between WKY and SD rats in avoidance acquisition. Although avoidance acquisition was not significantly different between SD and WKY rats, the general direction was for WKY rats to learn avoidance to a greater level than SD rats. Importantly, WKY rats still displayed more resistance to extinction training than SD rats. Thus, WKY rats had more persistent avoidance responding, despite the lack of strain differences in avoidance acquisition.

In addition to persistent responding during extinction, WKY rats and SD rats with hippocampal damage made more ITI responses, another type of non-reinforced responding. During the acquisition phase, lever-press responses during the first but not second or third minutes of ITI were higher for SD rats with hippocampal lesion and WKY rats, as compared to sham lesions and unoperated SD rats, respectively. During the extinction phase, ITI responses were higher in rats with hippocampal lesions (minute 1, not minutes 2 and 3) but not in WKY rats. One explanation for persistent avoidance responding during extinction training is that hippocampal lesions increase general activity, including lever-press responding. However, the lack of group differences during minutes 2 and 3 of the ITI suggests that this is not the case. The increase in ITI responding in WKY rats during the acquisition phase has been previously reported (Beck et al., 2010). WKY rats are not prone to higher general activity compared to SD rats given the behavioral inhibited temperament of WKY rats (Pare, 2000; McAuley et al., 2009), but the increased ITI responding during avoidance learning may be a result of enhanced stress behaviors demonstrated by WKY rats.

Depression and anxiety are commonly comorbid (Kessler et al., 2003), and a smaller hippocampus has been associated with both disorders (Sheline et al., 1996). In addition to the anxiety-like traits the WKY rat exhibits, it has previously been considered as a model of depression as it displays depressive-like behavior in the forced-swim test (Lopez-Rubalcava and Lucki, 2000). However, excessive avoidance is typically not associated with depression (Chase et al., 2010), but with anxiety (Mineka and Zinbarg, 2006). In those cases where a relationship between depression and avoidance is found, it is passive, not active, avoidance that is related to depression (Ottenbreit and Dobson, 2004). Moreover, avoidance symptoms in anxiety disorders may be the cause of depression in patients with comorbidity (Moitra et al., 2008). Therefore, the enhanced and persistent active avoidance observed in rats with hippocampal damage and in WKY rats is more consistent with a model of anxiety than depression.

In summary, previous human studies with PTSD patients have suggested that an abnormal hippocampus may be a risk factor for developing PTSD. Here, we present evidence that hippocampal damage facilitates the development of persistent avoidance responding, similar to symptoms of anxiety disorders in humans. Moreover, we provide support that an animal model of behavioral inhibition, a risk factor for anxiety disorders (Kagan et al., 1987) and associated with self-reported avoidance symptomology in combat veterans (Myers et al., 2012), has reduced hippocampal volume and impaired hippocampal synaptic plasticity. The present findings support the idea that hippocampal dysfunction due to impaired synaptic plasticity and reduced volume leads to abnormally persistent avoidance learning, which in and of itself is a risk factor to develop anxiety disorders.

## Conflict of Interest Statement

The authors declare that the research was conducted in the absence of any commercial or financial relationships that could be construed as a potential conflict of interest.

## References

[B1] American Psychiatric Association. (2013). Diagnostic and Statistical Manual of Mental Disorders, 5th Edn Arlington, VA: American Psychiatric Publishing

[B2] Bannerman D. M. Deacon R. M. Offen S. Friswell J. Grubb M. Rawlins J. N. (2002). Double dissociation of function within the hippocampus: spatial memory and hyponeophagia. Behav. Neurosci. 116, 884–901 10.1037/0735-7044.116.5.884 12369808

[B3] Barkus C. McHugh S. B. Sprengel R. Seeburg P. H. Rawlins J. N. Bannerman D. M. (2010). Hippocampal NMDA receptors and anxiety: at the interface between cognition and emotion. Eur. J. Pharmacol. 626, 49–56 10.1016/j.ejphar.2009.10.014 19836379 PMC2824088

[B4] Beck K. D. Jiao X. Pang K. C. Servatius R. J. (2010). Vulnerability factors in anxiety determined through differences in active-avoidance behavior. Prog. Neuropsychopharmacol. Biol. Psychiatry 34, 852–860 10.1016/j.pnpbp.2010.03.036 20382195

[B5] Black A. H. Nadel L. O’Keefe J. (1977). Hippocampal function in avoidance learning and punishment. Psychol. Bull. 84, 1107–1129 10.1037/0033-2909.84.6.1107 928572

[B6] Bremner J. D. (2001). Hypotheses and controversies related to effects of stress on the hippocampus: an argument for stress-induced damage to the hippocampus in patients with posttraumatic stress disorder. Hippocampus 11, 75–81; discussion 82–74. 10.1002/hipo.1023 11345127

[B7] Chase H. W. Frank M. J. Michael A. Bullmore E. T. Sahakian B. J. Robbins T. W. (2010). Approach and avoidance learning in patients with major depression and healthy controls: relation to anhedonia. Psychol. Med. 40, 433–440 10.1017/S0033291709990468 19607754

[B8] Clements K. M. Saunders A. J. Robertson B. A. Wainwright P. E. (2007). Spontaneously hypertensive, Wistar Kyoto and Sprague-Dawley rats differ in their use of place and response strategies in the water radial arm maze. Neurobiol. Learn. Mem. 87, 285–294 10.1016/j.nlm.2006.09.003 17056285

[B9] Clements K. M. Wainwright P. E. (2007). Spontaneously hypertensive, Wistar Kyoto and Sprague-Dawley rats differ in performance on a win-stay task and a conditioned cue preference task in the water radial arm maze. Behav. Brain Res. 183, 169–177 10.1016/j.bbr.2007.06.008 17664015

[B10] Ferguson S. A. Cada A. M. (2004). Spatial learning/memory and social and nonsocial behaviors in the spontaneously hypertensive, Wistar-Kyoto and Sprague-Dawley rat strains. Pharmacol. Biochem. Behav. 77, 583–594 10.1016/j.pbb.2003.12.014 15006470

[B11] Geisser S. Greenhouse S. W. (1958). An extension of Box’s results on the use of the F distribution in multivariate analysis. Ann. Math. Stat. 29, 885–891 10.1214/aoms/1177706545

[B12] Gilbertson M. W. Shenton M. E. Ciszewski A. Kasai K. Lasko N. B. Orr S. P. (2002). Smaller hippocampal volume predicts pathologic vulnerability to psychological trauma. Nat. Neurosci. 5, 1242–1247 10.1038/nn958 12379862 PMC2819093

[B13] Gilbertson M. W. Williston S. K. Paulus L. A. Lasko N. B. Gurvits T. V. Shenton M. E. (2007). Configural cue performance in identical twins discordant for posttraumatic stress disorder: theoretical implications for the role of hippocampal function. Biol. Psychiatry 62, 513–520 10.1016/j.biopsych.2006.12.023 17509537 PMC2768050

[B14] Gould E. Tanapat P. (1999). Stress and hippocampal neurogenesis. Biol. Psychiatry 46, 1472–1479 10.1016/S0006-3223(99)00247-4 10599477

[B15] Gould E. Tanapat P. McEwen B. S. Flugge G. Fuchs E. (1998). Proliferation of granule cell precursors in the dentate gyrus of adult monkeys is diminished by stress. Proc. Natl. Acad. Sci. U.S.A. 95, 3168–3171 10.1073/pnas.95.6.3168 9501234 PMC19713

[B16] Gould E. Woolley C. S. McEwen B. S. (1990). Short-term glucocorticoid manipulations affect neuronal morphology and survival in the adult dentate gyrus. Neuroscience 37, 367–375 10.1016/0306-4522(90)90407-U 2133348

[B17] Gurvits T. V. Shenton M. E. Hokama H. Ohta H. Lasko N. B. Gilbertson M. W. (1996). Magnetic resonance imaging study of hippocampal volume in chronic, combat-related posttraumatic stress disorder. Biol. Psychiatry 40, 1091–1099 10.1016/S0006-3223(96)00229-6 8931911 PMC2910907

[B18] Jiao X. Pang K. C. Beck K. D. Minor T. R. Servatius R. J. (2011). Avoidance perseveration during extinction training in Wistar-Kyoto rats: an interaction of innate vulnerability and stressor intensity. Behav. Brain Res. 221, 98–107 10.1016/j.bbr.2011.02.029 21376086 PMC3079807

[B19] Kagan J. Reznick J. S. Snidman N. (1987). The physiology and psychology of behavioral inhibition in children. Child Dev. 58, 1459–1473 10.2307/1130685 3691195

[B20] Kessler R. C. Berglund P. Demler O. Jin R. Koretz D. Merikangas K. R. (2003). The epidemiology of major depressive disorder: results from the National Comorbidity Survey Replication (NCS-R). JAMA 289, 3095–3105 10.1001/jama.289.23.3095 12813115

[B21] Kjelstrup K. G. Tuvnes F. A. Steffenach H. A. Murison R. Moser E. I. Moser M. B. (2002). Reduced fear expression after lesions of the ventral hippocampus. Proc. Natl. Acad. Sci. U.S.A. 99, 10825–10830 10.1073/pnas.152112399 12149439 PMC125057

[B22] Lopez-Rubalcava C. Lucki I. (2000). Strain differences in the behavioral effects of antidepressant drugs in the rat forced swimming test. Neuropsychopharmacology 22, 191–199 10.1016/S0893-133X(99)00100-1 10649831

[B23] McAuley J. D. Stewart A. L. Webber E. S. Cromwell H. C. Servatius R. J. Pang K. C. (2009). Wistar-Kyoto rats as an animal model of anxiety vulnerability: support for a hypervigilance hypothesis. Behav. Brain Res. 204, 162–168 10.1016/j.bbr.2009.05.036 19523988 PMC2723189

[B24] McEwen B. S. Albeck D. Cameron H. Chao H. M. Gould E. Hastings N. (1995). Stress and the brain: a paradoxical role for adrenal steroids. Vitam. Horm. 51, 371–402 10.1016/S0083-6729(08)61045-6 7483328

[B25] Messaoudi E. Ying S. W. Kanhema T. Croll S. D. Bramham C. R. (2002). Brain-derived neurotrophic factor triggers transcription-dependent, late phase long-term potentiation in vivo. J. Neurosci. 22, 7453–7461 12196567 10.1523/JNEUROSCI.22-17-07453.2002 PMC6757978

[B26] Mineka S. Zinbarg R. (2006). A contemporary learning theory perspective on the etiology of anxiety disorders: it’s not what you thought it was. Am. Psychol. 61, 10–26 10.1037/0003-066X.61.1.10 16435973

[B27] Moitra E. Herbert J. D. Forman E. M. (2008). Behavioral avoidance mediates the relationship between anxiety and depressive symptoms among social anxiety disorder patients. J. Anxiety Disord. 22, 1205–1213 10.1016/j.janxdis.2008.01.002 18282686

[B28] Morris R. G. Anderson E. Lynch G. S. Baudry M. (1986). Selective impairment of learning and blockade of long-term potentiation by an *N*-methyl-d-aspartate receptor antagonist, AP5. Nature 319, 774–776 10.1038/319774a0 2869411

[B29] Myers C. E. Vanmeenen K. M. Servatius R. J. (2012). Behavioral inhibition and PTSD symptoms in veterans. Psychiatry Res. 196, 271–276 10.1016/j.psychres.2011.11.015 22397911 PMC3361537

[B30] O’Donnell M. L. Elliott P. Lau W. Creamer M. (2007). PTSD symptom trajectories: from early to chronic response. Behav. Res. Ther. 45, 601–606 10.1016/j.brat.2006.03.015 16712783

[B31] Olton D. S. (1973). Shock-motivated avoidance and the analysis of behavior. Psychol. Bull. 79, 243–251 10.1037/h0033902 4633560

[B32] Ottenbreit N. D. Dobson K. S. (2004). Avoidance and depression: the construction of the cognitive-behavioral avoidance scale. Behav. Res. Ther. 42, 293–313 10.1016/S0005-7967(03)00140-2 14975771

[B33] Pang K. C. Jiao X. Sinha S. Beck K. D. Servatius R. J. (2011). Damage of GABAergic neurons in the medial septum impairs spatial working memory and extinction of active avoidance: effects on proactive interference. Hippocampus 21, 835–846 10.1002/hipo.20799 20865731 PMC3010529

[B34] Pardon M. C. Gould G. G. Garcia A. Phillips L. Cook M. C. Miller S. A. (2002). Stress reactivity of the brain noradrenergic system in three rat strains differing in their neuroendocrine and behavioral responses to stress: implications for susceptibility to stress-related neuropsychiatric disorders. Neuroscience 115, 229–242 10.1016/S0306-4522(02)00364-0 12401336

[B35] Pare W. P. (1989). Stress ulcer and open-field behavior of spontaneously hypertensive, normotensive, and Wistar rats. Pavlov. J. Biol. Sci. 24, 54–57 2726299 10.1007/BF02964537

[B36] Pare W. P. (1994). Open field, learned helplessness, conditioned defensive burying, and forced-swim tests in WKY rats. Physiol. Behav. 55, 433–439 10.1016/0031-9384(94)90097-3 8190758

[B37] Pare W. P. (2000). Investigatory behavior of a novel conspecific by Wistar Kyoto, Wistar and Sprague-Dawley rats. Brain Res. Bull. 53, 759–765 10.1016/S0361-9230(00)00362-2 11179840

[B38] Richmond M. A. Yee B. K. Pouzet B. Veenman L. Rawlins J. N. Feldon J. (1999). Dissociating context and space within the hippocampus: effects of complete, dorsal, and ventral excitotoxic hippocampal lesions on conditioned freezing and spatial learning. Behav. Neurosci. 113, 1189–1203 10.1037/0735-7044.113.6.1189 10636298

[B39] Schmaltz L. W. Giulian D. (1972). Faster acquisition of discriminated lever-press avoidance by hippocampectomized rats. J. Comp. Physiol. Psychol. 81, 149–154 10.1037/h0033325 5074302

[B40] Servatius R. J. Jiao X. Beck K. D. Pang K. C. Minor T. R. (2008). Rapid avoidance acquisition in Wistar-Kyoto rats. Behav. Brain Res. 192, 191–197 10.1016/j.bbr.2008.04.006 18501974

[B41] Sheline Y. I. Wang P. W. Gado M. H. Csernansky J. G. Vannier M. W. (1996). Hippocampal atrophy in recurrent major depression. Proc. Natl. Acad. Sci. U.S.A. 93, 3908–3913 10.1073/pnas.93.9.3908 8632988 PMC39458

[B42] Slomianka L. West M. J. (2005). Estimators of the precision of stereological estimates: an example based on the CA1 pyramidal cell layer of rats. Neuroscience 136, 757–767 10.1016/j.neuroscience.2005.06.086 16344149

[B43] Villarreal G. Hamilton D. A. Petropoulos H. Driscoll I. Rowland L. M. Griego J. A. (2002). Reduced hippocampal volume and total white matter volume in posttraumatic stress disorder. Biol. Psychiatry 52, 119–125 10.1016/S0006-3223(02)01359-8 12114003

[B44] Wang Z. Neylan T. C. Mueller S. G. Lenoci M. Truran D. Marmar C. R. (2010). Magnetic resonance imaging of hippocampal subfields in posttraumatic stress disorder. Arch. Gen. Psychiatry 67, 296–303 10.1001/archgenpsychiatry.2009.205 20194830 PMC2848481

[B45] Yoder R. M. Pang K. C. (2005). Involvement of GABAergic and cholinergic medial septal neurons in hippocampal theta rhythm. Hippocampus 15, 381–392 10.1002/hipo.20062 15630696

